# Tubulin and Tau: Possible targets for diagnosis of Parkinson’s and Alzheimer’s diseases

**DOI:** 10.1371/journal.pone.0196436

**Published:** 2018-05-09

**Authors:** Mohamed Salama, Ali Shalash, Alshimaa Magdy, Marianne Makar, Tamer Roushdy, Mahmoud Elbalkimy, Hanan Elrassas, Passent Elkafrawy, Wael Mohamed, Mohamed B. Abou Donia

**Affiliations:** 1 Medical Experimental Research Center (MERC), Faculty of Medicine, Mansoura University, Mansoura, Egypt; 2 Toxicology Department, Faculty of Medicine, Mansoura University, Mansoura, Egypt; 3 Department of Neurology, Faculty of Medicine, Ain Shams University, Cairo, Egypt; 4 Biochemistry Department, Faculty of Medicine, Mansoura University, Mansoura, Egypt; 5 Okasha Institute of Psychiatry, Faculty of Medicine, Ain Shams University, Cairo, Egypt; 6 Faculty of Science, Menoufia University, Shebeen Elkoum, Egypt; 7 Department of Pharmacology, Faculty of Medicine, Menoufia University, Shebeen Elkoum, Egypt; 8 Basic Medical Science Department, Kulliyyah of Medicine, International Islamic University Malaysia, Kuantan Pahang, Malaysia; 9 Department of Pharmacology and Cancer Biology, Duke University Medical Center, Durham, North Carolina, United States of America; University of Florida, UNITED STATES

## Abstract

Neurodegenerative diseases including Alzheimer’s disease (AD) and Parkinson’s disease (PD) are characterized by progressive neuronal loss and pathological accumulation of some proteins. Developing new biomarkers for both diseases is highly important for the early diagnosis and possible development of neuro-protective strategies. Serum antibodies (AIAs) against neuronal proteins are potential biomarkers for AD and PD that may be formed in response to their release into systemic circulation after brain damage. In the present study, two AIAs (tubulin and tau) were measured in sera of patients of PD and AD, compared to healthy controls. Results showed that both antibodies were elevated in patients with PD and AD compared to match controls. Curiously, the profile of elevation of antibodies was different in both diseases. In PD cases, tubulin and tau AIAs levels were similar. On the other hand, AD patients showed more elevation of tau AIAs compared to tubulin. Our current results suggested that AIAs panel could be able to identify cases with neuro-degeneration when compared with healthy subjects. More interestingly, it is possible to differentiate between PD and AD cases through identifying specific AIAs profile for each neurodegenerative states.

## Introduction

Parkinson’s disease (PD) and Alzheimer’s disease (AD) are the commonest neurodegenerative diseases with late diagnosis [1,2,3] and hence their devastating prognosis [1,2]. Clinical diagnosis of PD depends on several motor manifestations, which occur 10–20 years after the beginning of neuro-degeneration and loss of over 60% of dopaminergic neurons [4]. Similarly, pathological changes of AD develop over many years before manifestations of subtle cognitive impairment [5]. This gives a very low chance for development of effective treatment. Thus the discovery of early diagnostic biomarkers could be able to pave the way for discovering a new and effective neuro-protective strategies for these diseases [6].

As in most neurodegenerative states, PD is accompanied by neuronal damage in certain brain areas, namely substantia nigra and striatal dopaminergic system [7]. Likewise, AD is associated with neuronal loss that starts many years before the appearance of clinical manifestations [8]. Neuronal damage leads to the release of some neural proteins that pass through the damaged blood-brain barrier (BBB) and enter the systemic circulation. As these proteins are normally hidden from the body’s immune system, they will be received by humoral immunity as new antigens warranting the formation of autoantibodies (AIAs) against them. Therefore, these formed AIAs could be used as diagnostic peripheral biomarkers in several neurodegenerative diseases including PD and AD [9].

In the recent few years, there has been a growing interest and proof showing that humoral response may be involved in the pathogenesis of neurodegenerative diseases. Consequently, serum AIAs antibodies directed against different self-antigens may play a role in the pathophysiology of different neurodegenerative diseases [10–13]. Although several hypotheses have proposed the elevation of AIAs in patients’ sera, little attention has been paid for their use as biomarkers for diagnosis of PD [14–16]. The choice of AIAs panel depends on their role in the pathogenesis of a disease, hence the possibility of damage and release during the course of such disease [17,18]. One class of neuronal proteins that has a strong correlation to central nervous system (CNS) homeostasis and function is the microtubules group [19].

Microtubules (MTs) are polymers of α/β-tubulin heterodimers [20]. MTs are involved in many cellular functions e.g. cell division and cellular transport, through interaction with the so-called MT-associated proteins [21]. Therefore, it was hypothesized that neurons express their distinctive morphology through support by intra-neuronal MT-dependent networks [22]. It is noteworthy to mention that one important step in axonal degeneration is the dying-back phenomenon [23]. In this detrimental process, axonal degeneration begins distally back to the cell body in what is called *axon retraction model* [23]. The later seems to be initiated by disruption of neural cytoskeleton through defective MTs as reported previously [24,25] and confirmed by findings in several neurodegenerative conditions[26]. Based on the aforementioned notions, defects in tubulin (e.g. genetic abnormalities in its genes) could lead to several neurological states [27–29]. It is interesting to highlight that the dying back phenomenon plays an important role in PD neuronal death [30]. It was therefore hypothesized that there is a strong correlation between MTs and PD related protein such as leucine-rich repeat kinase 2 (LRRK2), synuclein, and Parkin [31,32].

Turning to another class of cytoskeletal proteins that has been deeply involved in regulation of MTs function, which is the Microtubule Associated Proteins (MAPs). Quite recently, considerable attention has been paid to study the role of Tau in neuro-degeneration [33]. Tau was reported to play a vital role in AD pathogenesis as well as in a group of other disorders that share tau protein abnormalities, hence, named collectively “tauopathies” [34, 35]. More interestingly, tau was proved to be involved in PD as well [36]. In the present study, we explore the possibility of using a panel of AIAs against two MTs related proteins; Tubulin (TUB) and Microtubule-Associated Protein Tau (TAU), as potential peripheral biomarkers for diagnosis of PD and AD.

## Materials and methods

### Patients recruitment

Twenty-six PD; 15 AD and 10 healthy subjects (as controls) were recruited for the current cross-sectional study from Ain Shams University, Movement Disorders Clinic and Okasha Institute of Psychiatry, Egypt. Subjects were exposed to comprehensive medical examination to confirm the staging and scoring of each disease. Controls were examined as well to exclude the possibility of any other neurodegenerative disorders.

Recruited PD patients were diagnosed according to the British Parkinson ‘s Disease Society Brain Bank criteria [37]. Staging of PD was done using the Unified Parkinson’s Disease Rating Scale (UPDRS), Hoehn and Yahr scale (H&Y), and Schwab and England scales (S & E) in “medication Off” and “On” states. Patients’ exclusion criteria included presence of dementia, atypical or secondary parkinsonism. Vis-à-vis AD Patients were diagnosed according to the NINCDS-ADRDA and DSM-IV criteria for dementia [38] and assessed using the Arabic version of the Montreal Cognitive Assessment (MoCA) test [39,40]

The study was approved by the Institutional Review Board (IRB) of Mansoura School of Medicine, and Ethical Committee of Faculty of Medicine, Ain Shams University. All participants signed informed consent prior to the enrolment in our study.

Blood samples were withdrawn from both control and case into anticoagulant-free tubes, then, sera were obtained by centrifugation of blood at 3000 RPM for 20 min at 4 C° then aliquoted and stored at -20 C° (for no more than 1 week) prior to their transfer to the Medical Experimental Research Center (MERC) of Mansoura University and stored at—80 ^0^C.

### Autoantibodies estimation

All antibodies have been assayed using a MyBioSource^TM^ enzyme-linked immunosorbent assay (*ELISA)* kits (San Diego, CA, USA). Serum concentrations of Microtubule-associated Protein Tau antibody (anti-MAPT) and Tubulin Alpha 1B Chain Antibody (TUBA1BA) were determined by quantitative sandwich ELISA (Cat. No. MBS9310801, MBS108941, respectively). Testing steps have been carried out according to the manufacturer’s provided protocols. The absorbance was read using an automatic microplate reader (Optica, Mikura Ltd, UK) at 450 nm. The sensitivity of the assay was 0.1 ng/ml.

### Data analysis

Data analysis was performed, using Statistical Package for Social Science (SPSS 20), to identify the ability of autoantibodies to separate cases (PD and AD) from controls. Continuous variables were compared using one-way ANOVA followed by post hoc analysis using the Bonferroni test.

To differentiate between controls, PD cases and AD cases, we performed the following analyses methods: individual correlation value for biomarkers, histograms and distribution curves. Correlations between serum levels of AIAs and clinical data of the studied groups using Pearson’s (for parametric data) and Spearman’s (for non-parametric data) coefficients tests.

## Results

### Demographic and clinical data of cases

Mean age of PD patients was 52.92 ± 11.98 years old (range 23–69), while the mean age of patients with AD was 71.88 ± 7.0 years old (range 61–81). Males were 50% of recruited subjects of both diseases. Table 1 summarizes demographic, clinical and serum autoantibodies data for both patients and control.

**Table 1:** Demographic, clinical and serum autoantibodies of patients with PD and AD diseases.

	PD Patients; Mean ± SD (n = 26)	AD patients; Mean ± SD (n = 15)	Controls; Mean ± SD (n = 10)
Age (years)	52.92 ± 11.98 (range 23–69)	71.88 ± 7.0 (range 61–81)	61.22 ± 5.4 (range 44–72)
DOI (years)	5.48 ± 3.03 (1–10)	4.47 ± 2.81 (1–9)	——————
AOO	47.44 ± 11.38 (22–63)	67.41 ± 6.19 (59–75)	——————
MOCA	——————	16.13±5.20	——————
H&Y off	3.33±1.19	——————	——————
H&Y on	1.54±1.15	——————	——————
S&E OFF	45.00±26.04	——————	——————
S&E ON	80.42±18.99	——————	——————
UPDRS I off	5.42±2.60	——————	——————
UPDRS II off	24.74±12.17	——————	——————
UPDRS III off	50.79±17.42	——————	——————
TUB AIAs	4.04 ± 0.30*	3,92 ± 0.26*	0.228 ± 0.50
Tau AIAs	3.93 ± 0.28*	7.72 ± 6.20*	0.45 ± 0.50

SD, standard deviation; H&Y, Hoehn and Yahr Scale; S&E, Schwab and England Scale, UPDRS, Unified Parkinson’s Disease Rating Scale, AIAs; autoantibodies

* = P < 0.001 compared to control.

### Autoantibodies levels

The results of both tested AIAs showed statistically significant elevation in both diseases (PD and AD) compared to controls (p< 0.001) (S1 Table). Post-Hoc analysis showed non-significant differences between PD and AD regarding serum AIAs of Tau (p = 0.069) and Tubulin (p = 1.00).

### Correlation values

From the data analysis, we could differentiate patients from controls where tubulin has the highest correlation to the cases (r = 0.629 for tubulin and r = 0.327 for tau).

**Fig 1:**
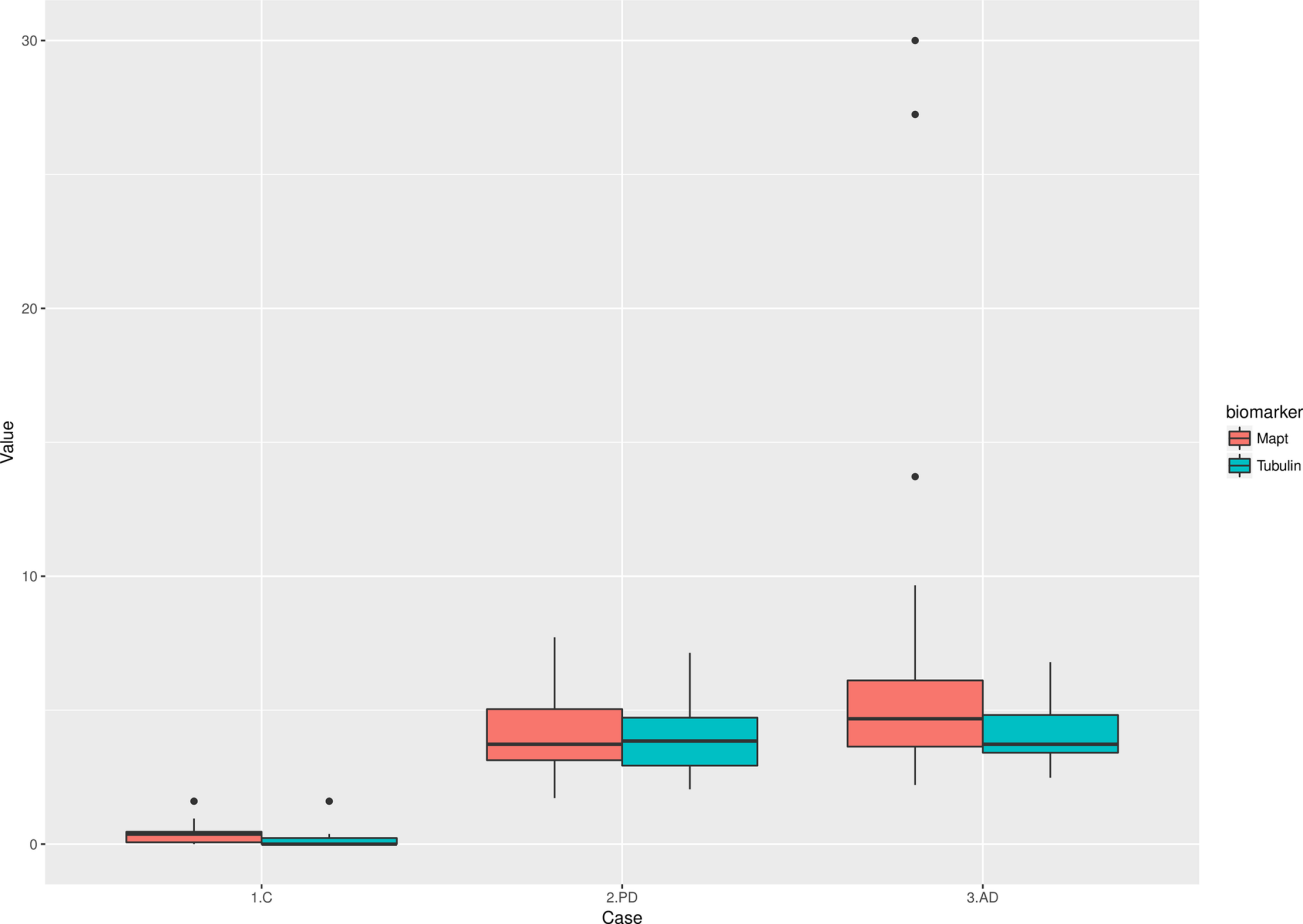
**Serum autoantibodies titers of Tubulin (A) and Tau (B) in investigated groups.** Higher levels of both AIAs were higher in patients of PD and AD compared to controls.

Tubulin AIAs level was a true differential factor between patients and control, wherein controls tubulin AIAs were not significant. In other words, when tubulin AIAs were detected in sera then it was an indication of a neurodegenerative disease as seen in “Fig 1”. Tau AIAs also showed the ability to differentiate patients from controls as seen in “Fig 1”. Serum Tau AIAs were significantly correlated to duration of illness (r = 0.563, p = 0.029) in patients with AD, and a trend for significance with MOCA scores (r = -0.490, p = 0.061), while tubulin serum AIAs did not show significant correlation. MOCA was correlated to the age of patients (r = 0.916, p = 0.013), and age of onset (r = -0.519, p = 0.039). Both AIAs did not show a significant correlation with UPDRS III off, H & Y off, and S& E off scales among PD patients.

### Histograms

**Fig 2:**
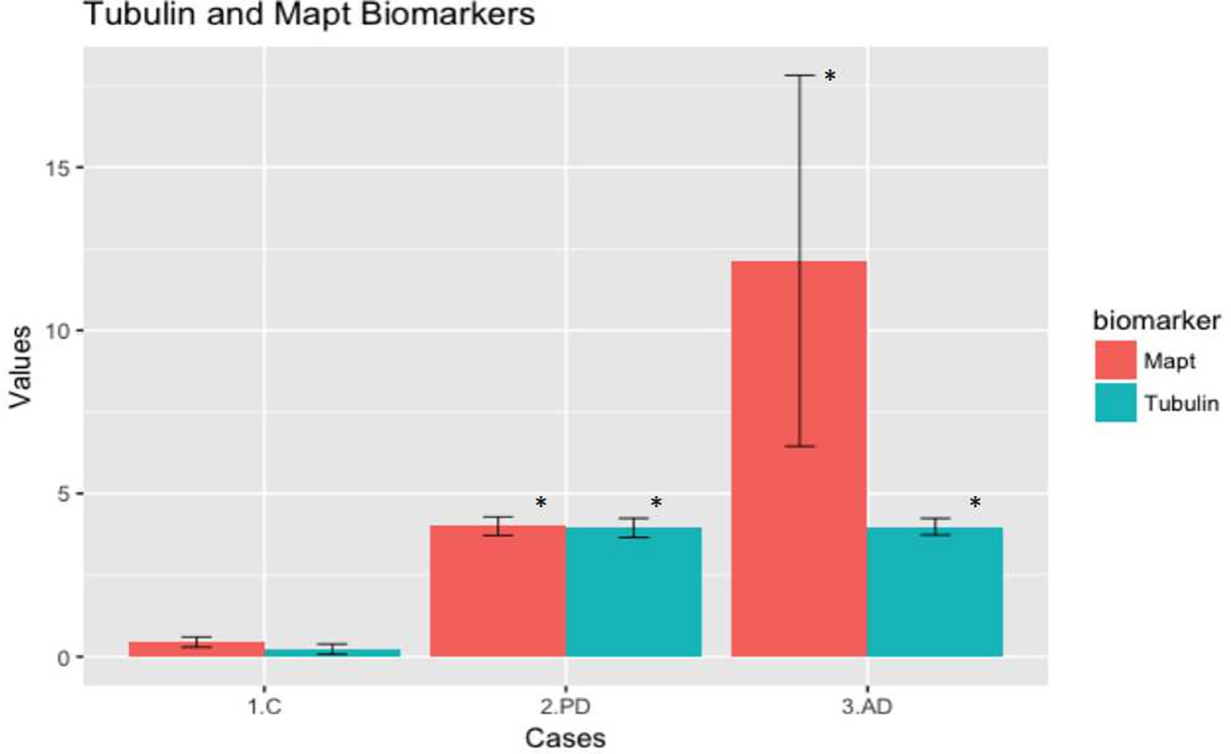
Histograms showing levels of autoantibodies in different groups (mean ± SEM). Tubulin AIAs show a significant elevation in PD and AD cases compared to control. On the other hands, Tau was elevated in both diseases with higher elevation in AD.

From the histograms “Fig 2”, we can easily separate patients from control using the biomarkers. Regarding differentiation between different diseases, most of PD patients showed a similar elevation for tubulin and tau AIAs levels. On the other hand, AD patients showed higher values of Tau AIAs compared to tubulin AIAs.

### Level distribution curves

**Fig 3:**
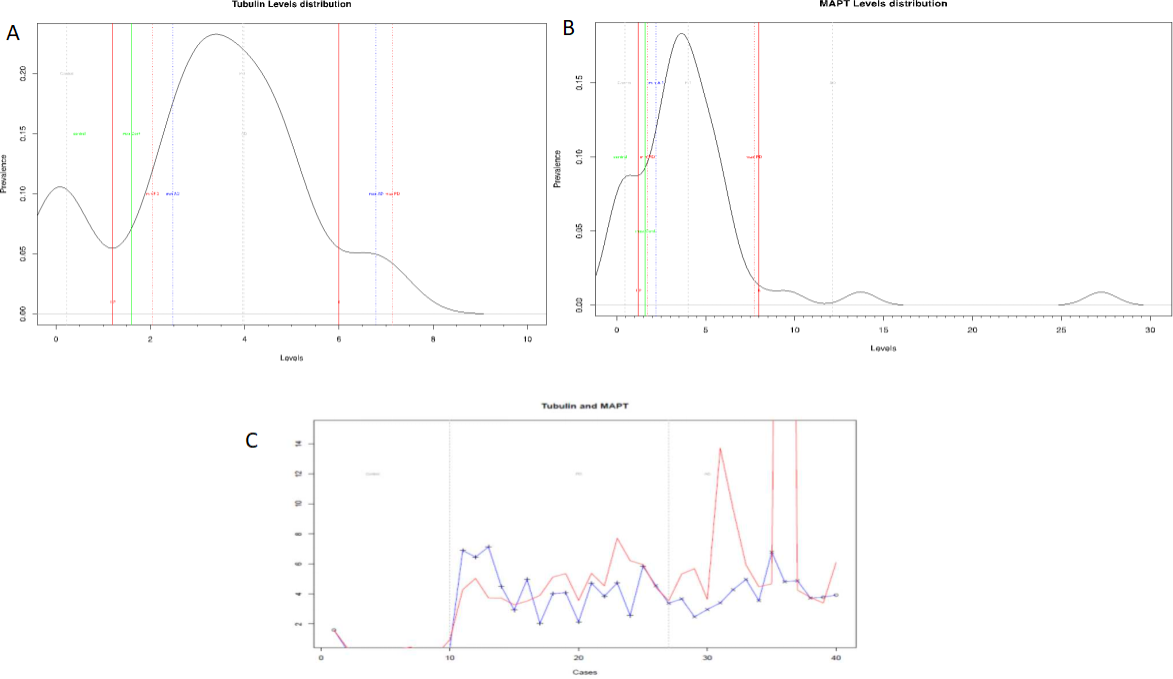
**Distribution curves of AIAs against tubulin (3A), tau (3B) and the combination of tubulin and tau (3C) in different groups.** Curves showed possible differentiation between cases and controls (3A and 3B) and the possibility of identifying profiles specific for PD and AD (Tubulin in blue and Tau in red) (3C).

Distribution curves showed possible classification of studied samples into three distinctive groups (based on the identification of peaks representing clusters of cases) as follows:

“Fig 3A” illustrates the distribution curve for tubulin AIAs. As can be seen from this figure, there are 3 groups: group 1: control—from 1 to 1.2; group 2 –from 1.2 to 6; and group 3 –from 6 upwards. Combining data from the curve to previous histogram graphs, group 2 should be AD and group 3 should be PD.

As shown in “Fig 3B”, tau distribution curve yielded 3 groups: group 1 –control—from 0 to 1.2; group 2 –from 1.2 to 8; and group 3 –from 8 upwards. Here AD range exceeds the curve and from previous histogram graphs, group 2 should be PD and group 3 should be AD.

Plotting data from both AIAs “Fig 3C” showed possible differentiation between cases and controls. Moreover, the curve shows the possibility of identifying different distribution profiles specific for PD and AD. (Tubulin in blue and Tau in red).

## Discussion

This paper is a modest contribution to the ongoing discussion on peripheral biomarkers for diagnosis of PD and AD. In the present report, we have evaluated a panel of two autoantibodies against MTs related proteins (Tubulin and Tau) as peripheral biomarkers for diagnosis of PD and AD. Our results showed that this small panel could differentiate patients from matched controls as evidenced by statistically significant elevation in the two autoantibodies levels among selected patients. Likewise, the panel showed a specific profile for each disease, hence, its benefit in differentiation between PD and AD.

There is a necessity for developing biomarkers that can help in early diagnosis of neurodegenerative diseases, including PD and AD [41]. Since conventional diagnostic tools could not identify in early stages, it is essential to use novel methods [6]. One promising approach is detecting serum autoantibodies against neural proteins involved in the pathogenesis of these disorders [42]. Quantification of these autoantibodies would be an easy, accessible and cost-efficient biomarker for neurodegenerative diseases [43]. The ideal biomarker should be sensitive and specific that it should identify every case compared to controls; meanwhile, it should also differentiate between similar diseases [44].

A challenge in choosing autoantibodies is to identify certain neural proteins that play important role in brain functions and affected in neurodegenerative diseases [45]. Our paper presents an innovated view of the two neural proteins related to microtubules that are deeply involved in the maintenance of cell integrity and function in the brain. Tubulin is the main protein in MTs system [21], as such, it is predicted that in cases of neural damage, tubulin will be affected. More important, tubulin has been proved to be involved in degeneration processes in PD and was linked to PD inducing toxins and related proteins [30–32].

We have addressed not only Tubulin but also Tau. The latter is an associated protein with MTs, hence it compliments the function to tubulin [36] though being involved in many neurodegenerative diseases beginning from AD, tauopathy group of diseases and even PD [35]. This suggests different profiling of Tau compared to tubulin, which would serve for differentiating between different diseases when using both AIAs.

In this report, tubulin was elevated in both diseases (PD and AD), which was anticipated based on the role of MTs in the stabilization of axonal cytoskeleton and neuronal connections [46]. Carletti et al. reported that gluta-thionylation of microtubules (e.g. tubulin) is the initiatory step for oxidative stress-induced damage of the neuronal cytoskeleton, hence, the dying back pattern of axonal degeneration [47]. Niwa et al confirmed the same later where they showed severe neuronal damage in cases of tubulin mutations [48].

The second estimated AIA, (tau), was elevated in both diseases, however, the higher values of tau AIAs were in AD cases compared to PD, which is justifiable by the pivotal role played by tau in AD pathogenesis [33]. More recently, tau levels showed to be high in CSF and sera of AD patients [49–50]. Additionally, we found that serum tau AIA was related to disease duration and MOCA scores (trend for significance) in AD patients. This is in line with earlier studies reporting a significant correlation of serum tau AIAs with cognitive functions [50–52]. The elevation of tau autoantibodies in PD patients has also been reported before [45], which may shed light on the plausible role of tau proteins in PD pathogenesis.

It is important to notice that despite the reported non-significant levels of AIAs between AD and PD, both diseases exerted different profiles, which could serve as a differentiation tool between them. To our knowledge, this the first study to report the qualitative changes between levels of AIAs. As can be seen in histograms, PD pattern showed a similar elevation in both AIAs, whereas, AD showed a higher elevation in tau AIAs compared to tubulin (although both AIAs were significantly higher than control). It is noteworthy that using distribution curves in biomarkers study is more suitable since the arithmetic mean is not a robust statistic tool to be applied for distribution studies, but is more suitable for deriving central tendency. This is mainly due to the fact that it could be affected by the variations of the values in the sample (either too small or too large). Distribution curves in our case showed a more differentiating pattern for cases and each disease.

The present findings of increased IgG against tubulin and tau in AD and PD patients show that both proteins could be involved in the pathogenesis of these diseases. A possible interpretation is that these AIAs represent a “*stable signature*” of neuronal damage in PD and AD [9]. On the other hand, the increase in AIAs—specifically against tubulin and tau could be explained by abnormal stimulation of immune system in the course of diseases [10] or a coexistence of immune-mediated polyneuropathy and neuro-degenerative disorders (PD and AD in our current work) [53]. It is claimed that combination of previous roles may be played by AIAs: earlier they are formed as a result of released proteins into systemic circulation through the pathway of neurodegenerative process, later, these AIAs begin to attack brain tissues leading to more neural damage [9].

Since AIAs represent an immunological memory of damaged proteins, they could be validated as "biomarkers of effect/injury" [54]. A question that could be raised here, is the value of such biomarkers for a clinician seeking early diagnosis, and when could they be applied. The answer to this question comes from a better understanding of most neurodegenerative disorders’ pathogenesis, where environmental exposure to risk factors interact actively with genetics to develop most of these disorders e.g. PD [55]. Since the exposure to environmental agents takes a long time until developing overt damage that can be clinically manifested, a validated biomarker of injury will be very useful as a detector of early exposure in the high-risk population. It is important to notice that the threshold of damage -needed to elicit an immune response, hence, the formation of AIAs- is much lower than the intensity of neuronal loss leading to overt clinical symptoms of the disease [9]. This may be the motive beyond the recommendations issued by Alzheimer's Association and the Alzheimer's Drug Discovery Foundation in 2012 advocating the need for peripheral, non-invasive blood-based biomarker that can diagnose the disease earlier than clinical signs [56]. We believe that AIAs could detect earlier brain damages years before clinical manifestations.

## Conclusion

To conclude, our panel -composed of two AIAs- was able to diagnose PD and AD (i.e. differentiating cases from controls). In addition, profiling different diseases seem possible. The profile of elevated AIAs in PD was different from AD with a possibility of individualization, hence specificity in the diagnosis of various neurodegenerative disorders. Needless to say, the main limitation of the current report is the small number of studied cases. We believe that recruiting a larger number of cases will, certainly, confirm our data. Nevertheless, our findings present the first report on using tubulin and tau AIAs as peripheral biomarkers for diagnosis of PD or AD.

## Supporting information

S1 Table Table showing individual AIAs levels in patients and controls. Dataset supporting the individual cases and controls values were uploaded to the Journal submission system.
(XLSX)
